# Measures taken to alleviate the impact of COVID‐19 outbreak on surgical patients

**DOI:** 10.1002/mco2.24

**Published:** 2020-09-03

**Authors:** Xiang‐Song Wu, Xu‐An Wang, Xu‐Heng Sun, Tai Ren, Li Zhao, Lin Shen, Yu‐Rong Gong, Yan‐Fei Xu, Shuai Huang, Ping Dong, Wei Gong, Xue‐Feng Wang, Ying‐Bin Liu

**Affiliations:** ^1^ Department of General Surgery Xinhua Hospital Shanghai Jiao Tong University School of Medicine Shanghai China; ^2^ Biliary‐pancreatic Surgery Department Renji Hospital Affiliated to Shanghai Jiao Tong University School of Medicine Shanghai China

The coronavirus disease 2019 (COVID‐19) outbreak has brought numerous challenges to the public health system since December 2019. Chinese government, hospitals, and doctors have made tremendous efforts to restrain the pandemic. All patients with fever or pneumonia of undetermined origin or suspected COVID‐19 infection are being transferred to COVID‐19‐designated hospitals for diagnosis and quarantine. To confront the massive demand of human and medical resources, a large number of medical professionals from various specialties have been voluntarily grouped together to support the front line against COVID‐19,^1^ and medical supplies are centralized in relevant departments. All these strict measures were taken as earlier as possible and had showed effectiveness in controlling the rapid spread of COVID‐19. As the number of new infections has been declining since February, we have achieved tremendous success in containing COVID‐19 infection and transmission in China and our experience of battling against this epidemic is valuable for the rest of the world.^2^

At the same time, the outbreak exerted tremendous impacts on surgical patients in China. Elective surgeries dropped by 70‐95% in Chinese hospitals during the epidemic (Figure 1A).^3^ The shortage of medical resources such as personal protective equipment and blood, and the strict administrative policy for preventing infection are the main reasons. In addition, due to the possible person‐to‐person infection in hospitals, surgical patients refrained from coming to hospital to seek medical care. However, certain surgical patients, such as acute abdomen, digestive tumors, cholelithiasis, gastrointestinal bleeding, and abdominal injury, are likely to face worse outcomes without timely surgery. Therefore, another issue arose as how to provide safe and high‐quality surgical care to patients during the COVID‐19 outbreak. In this regard, Chinese hospitals have taken following measures based the protocols from the National Health Commission of China and experience learned from the fight against SARS.^4^

**FIGURE 1:**
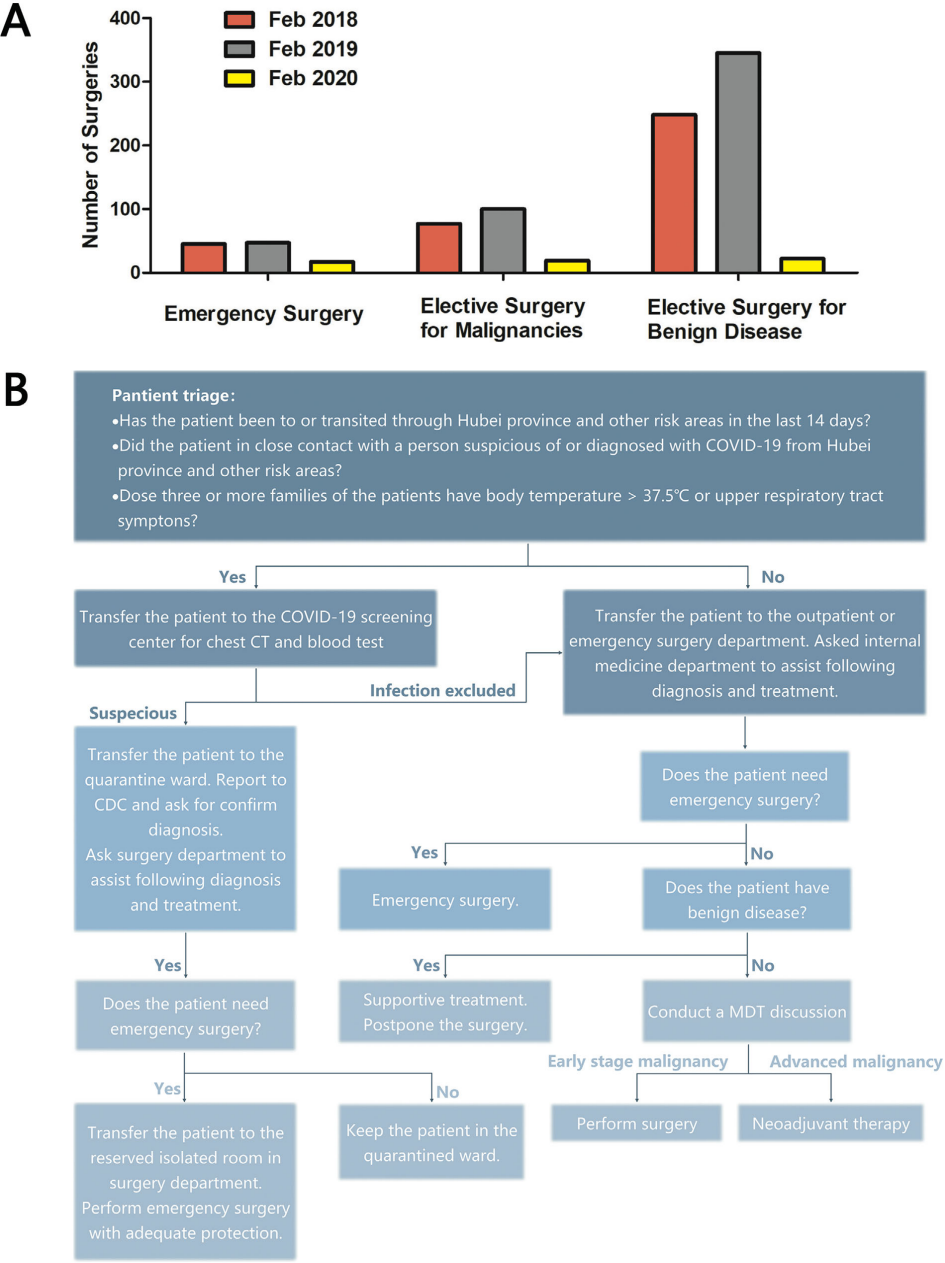
The outbreak exerted tremendous impacts on surgical patients and multiple measures were taken to prevent the COVID‐19 transmission in Xinhua Hospital. A, Surgery volume in Department of General Surgery, Xinhua Hospital in February 2018‐2020. B, Standard flowchart of surgical outpatients in Xinhua Hospital.

Let us consider Xinhua Hospital, an academic tertiary hospital with 4 million annual outpatient visits, as an example. Upon the outbreak, a standard flowchart (Figure 1B) that contains all the related information has been released and handed over to all staffs and preadmitted patients either in printed copies or through social media. First, every outpatient is requested to have body temperature measured and fill a form including following contents on patient triage: (a) a travel history especially to those outbreak areas within last 14 days, (b) any possible contacts with suspected or confirmed COVID‐19‐infected patients, and (c) symptoms of the upper respiratory tract, running nose, and/or diarrhea. Anyone who meets one of the above‐mentioned criteria would be requested to visit the newly established facility named the COVID‐19 Screening Center, which is an independent unit detached from the main medical buildings and dedicated to initial screen, identify, and contain the infection. Second, patients are encouraged to visit hospitals without unnecessary companion; only one patient is allowed in each consulting room during consultation or upon physical examination; to reduce the volume of unnecessary hospital visiting, online medical consultation and patient follow‐up are available by mobile phones and computers; over‐the‐counter medication are provided via e‐commerce platform. All surgeons and nurses wear white coats, medical masks, and medical hats for self‐protection. Upgrade to respirators N95, protective grown, gloves, and goggles would be applied, if a patient suspected of COVID‐19 infection seeks surgical consultation.

In the surgical ward, we take multiple measures to prevent the COVID‐19 transmission. To avoid close contacts among patients, admitted patients are put in separate rooms. If patients have to share the same room, each individual patient must be kept apart with each other with a safe distance of 1.5 meter. Each surgical ward is required to reserve one or two rooms for the isolation of any suspected COVID‐19 case. Once diagnosed with COVID‐19, the patient will be transferred to a designated medical center, which has the capabilities in dealing with contagious disease and performing surgical procedures. In Shanghai, all patients confirmed of COVID‐19 infection receive their treatment in Shanghai Public Health Clinical Center, which was built in 2004 after the SARS epidemic to provide specialized health care to patients with contagious disease.

Emergency surgeries are maintained and elective surgeries are conducted only when necessary. In life‐threatening conditions, the operation will be performed even if the patients are diagnosed with or suspected of COVID‐19 infection. Risk classification and high‐level protection are required in such a case, including hand hygiene, surgical mask, medical cap and clothes, disposable medical gloves for every staff and medical shoe covers, single‐use isolation gown, N95 mask, and other protections for more risky situation.^5^ For patients with benign diseases and no obvious symptoms, elective surgeries are postponed. For patients with malignant diseases, a multidisciplinary team (MDT) conducts an online discussion to decide the therapeutic strategy after taking fully consideration of tumor's biological behavior, clinical TNM staging, and the efficacy of neoadjuvant therapy. For patients with early‐stage digestive malignancy, surgeries are recommended after eliminating the possibility of COVID‐19 infection. As COVID‐19 is likely to spread through aerosol, procedures such as endoscopic examinations have been suspended for their risk of generating aerosol. For patients need pathological diagnosis by endoscopic ultrasound (EUS) biopsy before further therapy, the MDT team makes the clinical diagnosis and therapeutic strategy based on imaging findings and blood test.

Another issue is the psychological effect in cancer patients. The obliged self‐isolation can trigger various stressors, including prolonged duration, fears of infection, frustration, boredom, and inadequate information. During the outbreak, cancer patients might feel more frustrated because of increased chance of potential infection during hospital visiting, delayed diagnosis and treatment, and fail to transferring to a referred medical center located in another city due to the citywide lockdown. To relieve these psychological effects, medical institutions across China have opened online platforms to provide psychological counseling services. Moreover, to recover confidence and overcome fear for cancer patients, the government updates daily data of COVID‐19 outbreak to keep information transparent through social media and conventional outlets; hospitals and medical centers have released guidelines for outpatients and inpatients protection. Surgeons and oncologists provide counseling through online platforms for those patients hesitated to go to hospital.

The COVID‐19 infection poses significant challenge to health system worldwide. The outbreak placed a greater demand on the quality of clinical care, especially in surgery, where both safety and efficiency should be guaranteed. Hospitals are expected to take systematic measures to prevent infection in outpatients, wards, and operative theatre, while providing necessary medical services. Moreover, cancer patients might be easily neglected during an epidemic when the infection control is the focus. As the COVID‐19 infections has been declining for months in China, specific measures we are taking now to alleviate the impact of potential infection risk of COVID‐19 has also been simplified. Although the current condition of COVID‐19 disease has improved significantly and the restriction of raveling is relieving in China, these measures we took to prevent COVID‐19 infection in surgical patients during the hardest time are still of great meaning in medical management in hospitals. Government, hospitals, and surgeons should recognize such effects and make plans to alleviate any potential consequences.

## CONFLICT OF INTEREST

The authors declare no conflict of interest.
